# Unstable microhabitats (merocenoses) as specific habitats of Uropodina mites (Acari: Mesostigmata)

**DOI:** 10.1007/s10493-013-9659-9

**Published:** 2013-03-29

**Authors:** Agnieszka Napierała, Jerzy Błoszyk

**Affiliations:** Department of General Zoology, Faculty of Biology, Adam Mickiewicz University, ul. Umultowska 89, 61-614 Poznan, Poland

**Keywords:** Uropodina, Community structure, Soil, Decayed wood, Bird nests, Mammal nests

## Abstract

Unstable microhabitats (merocenoses)—such as decayed wood, ant hills, bird and mammal nests—constitute an important component of forest (and non-forest) environments. These microhabitats are often inhabited by specific communities of invertebrates and their presence increases the total biodiversity. The primary objective of the present study was to compare communities of Uropodina (Acari: Mesostigmata) inhabiting soil and unstable microhabitats in order to explore the specificity of these communities and their importance in such ecosystems. Uropodine communities inhabiting merocenoses are often predominated by one or two species, which constitute more than 50 % of the entire community. Many species occur commonly in particular merocenoses, but are absent or rare in soil and litter, for example, *Allodinychus flagelliger*, *Metagynella carpatica*, *Oplitis alophora*, and *Phaulodiaspis borealis*. The biology of Uropodina inhabiting unstable microhabitats is modified by the adaptations required for living in such habitats. Mites associated with merocenoses developed special dispersal mechanisms, such as phoresy, which enable them to migrate from disappearing environments. Communities of Uropodina in soil and litter predominately consisted of species which reproduce parthenogenetically (thelytoky), whereas in merocenoses bisexual species prevail.

## Introduction

Unstable microhabitats (merocenoses), such as decayed wood, ant hills, bird nests and mammal nests, are often scattered, small and ephemeral. As opposed to soil and litter, merocenoses have different environments in terms of the food, physio-chemical, and microclimatic conditions. Merocenoses are characterized by higher and relatively stable humidity during the year. Humidity is highly significant for mites from the suborder Uropodina (Acari: Mesotigmata) because mesohigrophilic species constitute the majority of these mites (Athias-Binche and Habersaat 1988; Krištofík et al. 1993; Błoszyk et al. 2001, 2004; Błoszyk and Bajaczyk 1999). Decayed wood, ant hills, bird nests, and mammal nests are important components of natural ecosystems—both forest and non-forest open environments, such as meadows, xerophilous grasses, and peat-bogs. Unstable microhabitats are often inhabited by specific communities of invertebrates, thus increasing the total biodiversity of the environment (Krištofík et al. 1993; Gwiazdowicz et al. 2000; Gwiazdowicz and Sznajdrowski 2000; Błoszyk et al. 2003a; Bajerlein and Błoszyk 2004; Gwiazdowicz and Klemt 2004; Gwiazdowicz and Kmita 2004).

The specific characteristics of merocenoses are favorable only for species with special reproduction and dispersal abilities, enabling them not only to colonize and populate these microhabitats, but also to escape from the vanishing habitat when the food resources become limited, to find a new suitable habitat (Athias-Binche 1984, 1993, 1994; Faasch 1967). Uropodina use representatives of various orders of insects and centipedes as carriers (Mašán 2001). The carrier organisms enable the mites to cover distances between merocenoses and find microhabitats with a suitable microclimate and sufficient food resources. Phoretic deutonymphs of Uropodina have a special anal apparatus (pedicel), which enables a mite to stick to the carier’s body (Athias-Binche 1984; Błoszyk et al. 2006b). The structural complexity of the anal apparatus shows that Uropodina have probably had this ability for a very long time and no other group of mites has adapted to phoresy (Athias-Binche 1984).

Very few studies in the acarological literature adduce data about habitat preferences and assimilation abilities of Uropodina species, both to living in soil and specific merocenoses (Athias-Binche 1979, 1982a, b, 1983; Błoszyk (1992); Huţu 1993; Błoszyk 1999; Mašán 2001). The scant evidence obtained so far suggests that the biology of these species is modified by adaptation to living in each of these habitats. The observations carried out by Błoszyk et al. in different habitats in Poland have revealed differences in species composition and community structure of mites from the suborder Uropodina. The differences are most evident in the case of unstable microhabitats (Błoszyk 1980, 1983, 1985, 1999; Bloszyk and Olszanowski 1985a, b, 1986; Błoszyk et al. 2003a; Napierała et al. 2009). The reproductive strategies also appear to differ between the two community types (Błoszyk et al. 2004).

The studies on communities in unstable microhabitats help to understand the biology and ecology of uropodine mites, and offer an insight into functioning of such ecosystems. However, most papers published so far are based on rather small data sets and have a local character, which means that they deal with one type of merocenoses. Many papers have been published in local journals and are not in English, which makes them inaccessible for many potential readers (Błoszyk 1985, 1990; Błoszyk and Olszanowski 1985a, b, 1986; Błoszyk and Bajaczyk 1999; Gwiazdowicz et al. 2000, 2005, 2006; Bajerlein and Błoszyk 2004; Gwiazdowicz and Klemt 2004; Gwiazdowicz and Kmita 2004; Błoszyk and Gwiazdowicz 2006). The aim of the present study is to compare the communities of Uropodina inhabiting soil and unstable microhabitats to establish the features common for all merocenoses and what makes them different from soil environment. None of the studies published hitherto is based on such a large amount of material, collected during such a long period of time.

The main hypothesis is that in merocenoses there are one or two species that dominate the community, whereas in soil there is no strong dominance of one species. The second hypothesis is that parthenogenetic species prevail in soil, whereas bisexual species dominate in unstable microhabitats, depending on the variation in stability and size of these environments. The third hypothesis postulated here is that the presence of microhabitats in ecosystems increases the total biodiversity of uropodine fauna in such environments.

## Materials and methods

### Mite collection and extraction

The material for this study has been collected since 1951 in different parts of Poland (most samples come from Wielkopolska, Poland). Every month between 2001 and 2004 the soil and dead wood samples were collected in three nature reserves—Huby Grzebieniskie, Bytyńskie Brzęki, and Brzęki przy Starej Gajówce. They belong to a forest complex at about 25 km west-north-west from Poznań (for a detailed description see Napierała et al. 2009).

The soil was sampled quantitatively (core samples of 30 cm^2^ surface and 10 cm deep) and qualitatively (sieve samples). The mites were also collected from 0.5–0.8 l samples of different types of dead wood (rotten trunks, logs, and stumps). The mites were extracted with Tullgren funnels for ca. 4–6 days (depending on the level of moisture), and preserved in 75 % alcohol. Both permanent and temporary microscope slides were made (mounted in Hoyer’s medium), and the specimens were identified with the keys in Kadite and Petrova (1977), Evans and Till (1979), Karg (1989), Błoszyk (1999), and Mašán (2001). The 16,323 samples were collected and deposited in a soil-fauna database (Natural History Collections, Faculty of Biology AMU, Poznań); 13,996 samples were collected from soil, 978 from dead wood, 238 from tree holes, 233 from mammal nests, 836 from bird nests, and 42 from ant hills.

### Data analysis

The zoocenological analysis of Uropodina communities is based on the indices of the dominance and frequency. The following classes were used (Błoszyk 1999):

- Dominance: D5, eudominants (>30 %), D4, dominants (15.1–30.0 %), D3, subdominants (7.1–15.0 %), D2, residents (3.0–7.0 %), and D1, subresidents (<3 %).
- Frequency: F5, euconstants (>50 %), F4, constants (30.1–50 %), F3, subconstants (15.1–30.0 %), F2, accessory species (5.0–15.0 %), and F1, accidents (<5 %).

The community similarity was calculated by means of the Marczewski-Steinhaus species similarity index: MS = c/(a + b − c), where c is the number of species present in both compared communities, and a and b stand for the total numbers of species in each community (Magurran 2004).

The differences between the average abundances in the merocenoses and soil were analysed with Kruskal–Wallis ANOVA and Dunn tests. The mean abundances of the selected dominant species of Uropodina in the soil and dead wood in the three nature reserves of Wielkopolska were analysed with Mann–Whitney U tests. All tests were calculated in STATISTICA 6.0 Pl.

## Results

The total number of Uropodina collected in the presented material is 74 species (Table 1): 68 species (108,737 specimens) were found in the soil, 51 (19,843 specimens) in dead wood, 34 (3,069 specimens) in tree holes, 30 (7,696 specimens) in mammal nests, 28 (7,741 specimens) in bird nests, and 12 (871 specimens) in ant hills (Table 2).

**Table 1:** List of Uropodina species found in the analysed material

Species	Total	Adult		Juvenile		
		Female	Male	Deutonymph	Protonymph	Larva
*Trachytes aegrota* (C. L. Koch, 1841)	32,495	18,671	3	10,619	2,414	788
*Trachytes irenae* (Pecina, 1970)	11,450	3,012	4,501	3,176	588	173
*Trachytes lamda* (Berlese, 1903)	449	206	7	156	71	9
*Trachytes minima* (Trägårdh, 1910)	559	285	222	38	9	5
*Trachytes montana* (Willmann, 1953)	21	20		1		
*Trachytes pauperior* (Berlese, 1914)	7,683	3,396	31	2,326	1,257	673
*Trachytes splendida* (Hutu, 1973)	8	4	4			
*Polyaspinus cylindricus* (Berlese, 1916)	1,345	818		322	133	72
*Polyaspinus schweizeri* (Hutu, 1976)	11	7	4			
*Apionoseius infirmus* (Berlese, 1887)	1,567	429	345	652	138	3
*Polyaspis patavinus* (Berlese, 1881)	328	108	71	114	30	5
*Polyaspis sansonei* (Berlese, 1916)	165	30	33	60	24	18
*Uroseius hunzikeri* (Schweizer, 1922)	2		2			
*Iphidinychus gaieri* (Schweizer, 1961)	7	4		2	1	
*Discourella modesta* (Leonardi, 1889)	335	296	1	28	8	2
*Trematurella elegans* (Kramer, 1882)	700	263	281	132	18	6
*Oodinychus karawaiewi* (Berlese, 1903)	8,595	2,595	2,875	1,895	1,139	91
*Oodinychus obscurasimilis* (Hirschmann et Z.-Nicol, 1961)	432	184	214	25	8	1
*Oodinychus ovalis* (C. L. Koch, 1839)	21,586	5,997	6,022	4,645	3,760	1,162
*Oodinychus spatulifera* (Moniez, 1892)	796	373	339	82		2
*Iphiduropoda penicillata* (Hirschmann et Z.-Nicol, 1961)	35	21	11	3		
*Leiodinychus orbicularis* (C. L. Koch, 1839)	2,911	998	816	902	171	24
*Pseudouropoda calcarata* (Hirschmann et Z.-Nicol, 1961)	56	27	21	7	1	
*Pseudouropoda structura* (Hirschmann et Z.-Nicol, 1961)	5	1	4			
*Pseudouropoda tuberosa* (Hirschmann et Z.-Nicol, 1961)	14	5	3	4	2	
*Pseudouropoda* sp.	215	118	36	52	9	
*Urodiaspis tecta* (Kramer, 1876)	8,989	6,702		1,516	585	186
*Urodiaspis stammeri* (Hirschmann et Z.-Nicol, 1969)	461	228	226	4	3	
*Urodiaspis pannonica* (Willmann, 1952)	1,976	1,252		522	147	55
*Olodiscus kargi* (Hirschamann et Z.-Nicol, 1969)	253	144	86	22	1	
*Olodiscus minima* (Kramer, 1882)	15,585	12,647	56	2,066	563	253
*Olodiscus misella* (Berlese, 1916)	757	609		133	7	8
*Neodiscopoma splendida* (Kramer, 1882)	2,741	940	1,241	396	153	11
*Cilliba cassidea* (Herman, 1804)	208	84	92	25	7	
*Cilliba cassideasimilis* (Błoszyk, Stachowiak, Halliday 2007)	1,458	471	587	255	105	40
*Cilliba erlangensis* (Hirschmann et Z.-Nicol, 1969)	104	84		4	16	
*Cilliba rafalskii* Błoszyk, (Stachowiak, Halliday 2007)	620	369		122	73	56
*Cilliba selnicki* (Hirschmann et Z.-Nicol, 1969)	120	50	66	2	2	
*Uroobovella fracta* (Berlese, 1916)	4	1	3			
*Uroobovella marginata* (C. L. Koch, 1829)	32	6	8	16	2	
*Uroobovella obovata* (Canestrini et Berlese, 1884)	145	74	53	18		
*Uroobovella pulchella* (Berlese, 1904)	3,908	1,687	96	1,241	692	192
*Uroobovella pyriformis* (Berlese, 1920)	2,618	1,077	879	546	104	12
*Uroobovella* sp.	23	15	6	2		
*Fuscouropoda appendiculata* (Berlese, 1910)	8	3	1	4		
*Allodinychus flagelliger* (Berlese, 1910)	298	64	40	136	56	2
*Phaulodiaspis advena* (Trägårdh, 1912)	1,063	227	213	509	105	9
*Phaulodiaspis borealis* (Sellnick, 1940)	3,229	939	763	1,403	118	6
*Phaulodiaspis rackei* (Oudemans, 1912)	1,483	458	556	377	85	7
*Uroplitella conspicua* (Berlese, 1903)	22	19	3			
*Uroplitella paradoxa* (Canestrini et Berlese, 1884)	22	18	4			
*Oplitis alophora* (Berlese, 1903)	6	4	1			1
*Oplitis wasmanni* (Kneissl, 1907)	1	1				
*Oplitis* sp.	5	4	1			
*Trachyuropoda coccinea* (Michael, 1891)	152	82	58	9	3	
*Trachyuropoda poppi* (Hirschmann et Z.-Nicol, 1969)	1		1			
*Trachyuropoda willmanni* (Hirschmann et Z.-Nicol, 1969)	17	2	4	9	2	
*Urotrachytes formicarius* (Lubbock, 1881)	22	7	14	1		
*Dinychura cordieri* (Berlese, 1916)	509	226	154	94	35	
*Uropolyaspis hamulifera* (Berlese, 1904)	20	1	2	15	2	
*Discourella* (?) *baloghi* (Hirschmann et Z.-Nicol, 1969)	999	349	336	287	24	3
*Uropoda italica* (Hirschmann et Z.-Nicol, 1969)	4	4				
*Uropoda orbicularis* (Muller, 1776)	584	62	8	490	24	
*Uropoda undulata* (Hirschmann et Z.-Nicol, 1969)	38	25	12		1	
*Nenteria breviunguiculata* (Willmann, 1949)	1,751	416	273	897	152	13
*Nenteria floralis* (Karg 1986)	2	1	1			
*Nenteria stylifera* (Berlese, 1904)	53	31	2	8	11	1
*Dinychus arcuatus* (Trägårdh, 1922)	408	150	185	59	14	
*Dinychus carinatus* (Berlese, 1903)	1,009	311	290	302	88	18
*Dinychus inermis* (C. L. Koch, 1841)	339	154	132	42	11	
*Dinychus perforatus* (Kramer, 1882)	3,481	1,120	1,361	785	197	18
*Dinychus woelkiei* (Hirschmann et Zirngiebl-Nicol, 1969)	495	100	122	208	65	
*Metagynella carpatica* (Balogh, 1943)	163	14	12	131	6	
*Protodinychus punctatus* (Evans, 1957)	1	1				
Total	147,957	69,101	23,794	37,897	13,240	3,925

**Table 2:** Occurrence of Uropodina in studied microhabitats

Species	Soil	DW	TH	NM	NB	AH	No. of habitats where species was found
*T. aegrota*	+	+	+	+	+	+	6
*Oo. karawaiewi*	+	+	+	+	+	+	6
*Oo. ovalis*	+	+	+	+	+	+	6
*Uro. pyriformis*	+	+	+	+	+	+	6
*Din. perforatus*	+	+	+	+	+	+	6
*T. irenae*	+	+	+	+		+	5
*A. infirmus*	+	+	+	+	+		5
*Po. patavinus*	+	+	+	+	+		5
*Tre. elegans*	+	+	+	+	+		5
*I. penicillata*	+	+	+	+	+		5
*L. orbicularis*	+	+	+	+	+		5
*Pseudouropoda* sp.	+	+	+	+	+		5
*Ur. tecta*	+	+	+	+	+		5
*Ol. minima*	+	+	+	+	+		5
*Uro. obovata*	+	+	+	+	+		5
*Din. arcuatus*	+	+	+	+	+		5
*Din. carinatus*	+	+	+	+	+		5
*Din. woelkiei*	+	+	+			+	4
*Uroobovella* sp.	+	+	+		+		4
*Oo. spatulifera*	+	+	+			+	4
*T. pauperior*	+	+	+	+			4
*Ur. pannonica*	+	+	+		+		4
*P. cylindricus*	+	+	+		+		4
*Ol. misella*	+	+	+	+			4
*Ne. splendida*	+	+	+	+			4
*Tr. coccinea*	+	+	+			+	4
*Ps. calcarata*	+	+		+		+	4
*U. orbicularis*	+	+		+	+		4
*N. breviunguiculata*	+	+		+	+		4
*T. montana*	+	+		+			3
*Dis. baloghi*	+	+	+				3
*Uro. pulchella*	+	+	+				3
*Po. sansonei*	+	+	+				3
*Oo. obscurasimilis*	+	+	+				3
*C. cassideasimilis*	+	+	+				3
*Dis. modesta*	+	+		+			3
*D. cordieri*	+	+			+		3
*Ph. rackei*	+	+		+			3
*Uro. marginata*	+	+			+		3
*Din. inermis*	+	+		+			3
*Urlop. paradoxa*	+		+			+	3
*T. lamda*	+	+					2
*T. minima*	+	+					2
*P. schweizeri*	+	+					2
*Ps. structura*	+				+		2
*Ps. tuberosa*	+	+					2
*Ur. stammeri*	+	+					2
*Ol. kargi*	+	+					2
*C. erlangensis*	+	+					2
*C. rafalskii*	+	+					2
*C. selnicki*	+				+		2
*Uropl. conspicua*	+					+	2
*Urop. hamulifera*	+				+		2
*Ph. advena*	+			+			2
*N. stylifera*	+	+					2
*Oplitis* sp.	+		+				2
*Al. flagelliger*		+			+		2
*C. cassidea*	+						1
*Urot. formicarius*	+						1
*U. undulata*	+						1
*F. appendiculata*	+						1
*Ne. splendida*	+						1
*Tr. willmanni*	+						1
*Ip. gaieri*	+						1
*Uro. fracta*	+						1
*Opl. wasmanni*	+						1
*Tr. poppi*	+						1
*U. italica*	+						1
*Pr. punctatus*	+						1
*M. carpatica*		+					1
*Opl. alophora*			+				1
*Uros. hunzikeri*				+			1
*Ph. borealis*				+			1
*N. floralis*					+		1
No. of species	68	51	34	30	28	12	

*Soil* soil and litter, *DW* dead wood, *TH* tree holes, *NM* mammal nests, *NB* bird nests, *AH* ant hills

**Fig. 1:**
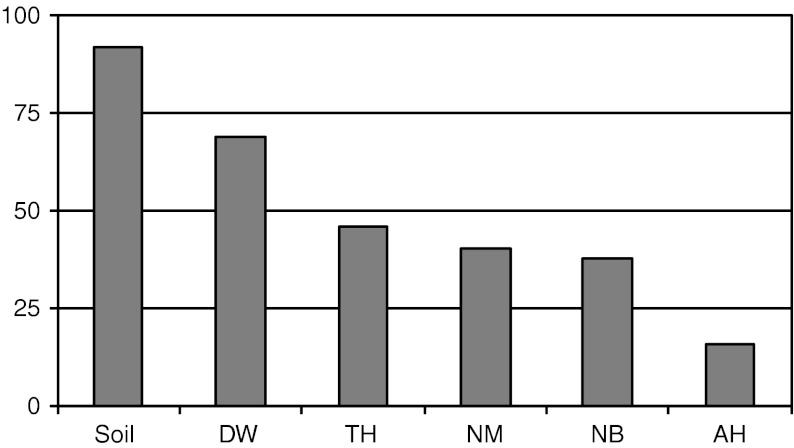
Percentage of species found in soil and various microhabitats with reference to the total number of species in Poland: *soil* soil and litter, *DW* dead wood, *TH* tree holes, *NM* mammal nests, *NB* bird nests, *AH* ant hills

In the analysed microhabitats, most species (69 % of the whole Polish fauna) occurred in dead wood, whereas the lowest number of species (16 %) was observed in ant hills. The other microhabitats contained similar percentages (38–46) of the Polish fauna of Uropodina (Fig. 1).

**Fig. 2:**
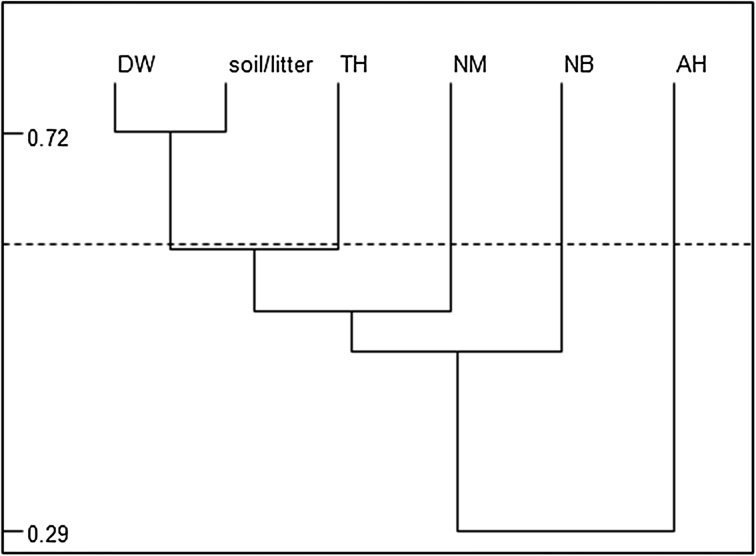
Similarity (S) of species composition of the communities of Uropodina in soil and in the analysed microhabitats: *soil* soil and litter, *DW* dead wood, *TH* tree holes, *NM* mammal nests, *NB* bird nests, *AH* ant hills

The bird nests and dead wood had the highest average number of mites, whereas ant hills had the lowest average number of mites. The most striking similarities in species composition (72 %) were found between the communities in the soil and the communities of Uropodina inhabiting the merocenoses of dead wood. The most distinct communities (29 % similarity) occurred in ant hills (Fig. 2).

### Species composition and community structure in analysed merocenoses

The highest frequency of Uropodina (>50 %) was observed in mammal nests, ant hills, and dead wood (Table 3). There were also significant differences in the average abundance of Uropodina in the analysed merocenoses (Table 4). The Uropodine mites were less frequent in bird nests—they have not been found in >85 % of the analysed nests. The highest average number of Uropodina (>30 specimens) per sample is in ant hills, dead wood, and mammal nests.

**Table 3:** Frequency and average number of Uropodina in soil and unstable microhabitats

	Soil	DW	TH	NM	NB	AH
No. of samples	13,996	978	238	233	836	42
Frequency of Uropodina (%)	41.4	50.5	44.1	61.4	14.6	57.1
Average no. of specimens per sample	7.7	38.9	12.4	33.0	9.3	39.9
95 % confidence interval	0.56	4.50	6.60	7.79	14.77	14.19

*Soil* soil and litter, *DW* dead wood, *TH* tree holes, *NM* mammal nests, *NB* bird nests, *AH* ant hills

**Table 4:** Pairwise comparison of average abundance of Uropodina in the analysed merocenoses and soil

	Ant hills	Dead wood	Tree holes	Mammal nests	Bird nests
Dead wood	ns				
Tree holes	ns	**			
Mammal nests	ns	ns	***		
Bird nests	***	***	***	***	
Soil	*	***	ns	***	***

Kruskal–Wallis ranks ANOVA (H = 330.94, *df* = 5, *P* < 0.001; n = 16,341) followed by Dunn’s test: * 0.01 < *P* < 0.05; ** 0.001 < *P *< 0.01; *** *P* < 0.001; *ns* not significant (*P* > 0.05)

The abundance of three most numerous species, i.e., *Trachytes aegrota*, *Oodinychus ovalis,* and *Oodinychus karawaiewi,* turned out to differ significantly in the analysed environments, but two other highly abundant species—*Uroobovella pyriformis* and *Dinychus perforatus*—were distributed evenly (Table 5).

**Table 5:** Pairwise comparison of average abundance of the dominant species of Uropodina in soil and the analysed merocenoses

Species	Comparisons														
	1–2	1–3	1–4	1–5	1–6	2–3	2–4	2–5	2–6	3–4	3–5	3–6	4–5	4–6	5–6
*T. aegrota* H = 450.51	*	***	***	***	***	ns	ns	ns	ns	ns	**	ns	***	ns	ns
*Oo. ovalis* H = 528.93	ns	ns	ns	***	***	ns	ns	***	ns	ns	***	***	***	***	ns
*Oo. karawaiewi* H = 27.83	ns	***	ns	ns	ns	ns	ns	ns	ns	***	***	**	ns	ns	ns
*Uro. pyriformis* H = 16.2	ns	ns	ns	ns	**	ns	ns	ns	ns	ns	ns	ns	ns	ns	ns
*D. perforatus* H = 8.38	ns	ns	ns	ns	ns	ns	ns	ns	ns	ns	ns	ns	ns	ns	ns

Kruskal–Wallis ranks ANOVA (all species: *df* = 5, *P* < 0.001; n = 16,323) followed by Dunn’s test: * 0.01 < *P* < 0.05; ** 0.001 < *P* < 0.01; *** *P* < 0.001; *ns* not significant (*P* > 0.05)

*1* soil, *2* ant hills, *3* mammal nests, *4* bird nests, *5* dead wood, *6* tree holes

#### Dead wood

This microenvironment is inhabited by most species of Uropodina: 51 species (Table 2). The most numerous and frequent species was *Oo. ovalis*, the second most numerous species was *Uro. pulchella* (Table 6). These two species constituted about 75 % of the whole community. *Metagynella carpatica* is one of those extremely rare uropodine species in Poland, and it occurred only in dead wood.

**Table 6:** Zoocenological analysis of dominance (classes D5-D1) and frequency (F5-F1) of the uropodine communities of the analysed merocenoses (see “Materials and methods” for a description of the classes)

Dominance		Frequency	
*Soil and litter*			
D5—eudominants	0	F5—euconstants	0
D4—dominants	*T. aegrota*—28.89 %	F4—constants	*T. aegrota*—35.63 %
D3—subdominants	*Ol. minima*—13.20 %	F3—subconstants	*Ol. minima*—26.81 %
	*T. irenae*—10.31 %		*Ur. tecta*—15.52 %
	*Oo. ovalis*—8.42 %		*T. pauperior*—15.37 %
	*Oo. karawaiewi*—7.18 %	F2—accesory species	*Oo. ovalis*—9.42 %
D2—residents	*T. pauperior*—6.99 %		*T. irenae*—6.36 %
D1—subresidents	62 species		*U. pannonica*—5.54 %
			*Din. perforatus*—5.00 %
		F1—accidents	61 species
*Dead wood*			
D5—eudominants	*Oo. ovalis*—56.38 %	F5—euconstants	0
D4—dominants	*Uro. pulchella*—18.60 %	F4—constants	*Oo. ovalis*—48.36 %
D3—subdominants	0	F3—subconstants	*T. aegrota*—20.45 %
D2—residents	*T. aegrota*—3.49 %		*Uro. pulchella*—16.87 %
	*Dis.* *baloghi*—3.05 %	F2—accesory species	*Ol. minima*—14.21 %
D1—subresidents	48 species		*Din. carinatus*—7.67 %
			*Ur. tecta*—5.11 %
		F1—accidents	46 species
*Tree holes*			
D5—eudominants	0	F5—euconstants	0
D4—dominants	*Oo. ovalis*—23.66 %	F4—constants	*Oo. ovalis*—39.50 %
	*Uro. pyriformis*—22.29 %	F3—subconstants	0
D3—subdominants	*Dis.* *baloghi*—11.65 %	F2—accesory species	*Uro. pyriformis*—13.03 %
	*T. aegrota*—9.69 %		*Dis.* *baloghi*—9.24 %
	*P*. *patavinus*—7.47 %		*T. aegrota*—8.82 %
D2—residents	*Din. carinatus*—5.74 %		*Din. carinatus*—7.56 %
	*Din. woelkei*—3.56 %		*Uro. pulchella*—7.14 %
	*Uro. pulchella*—3.17 %		*Ur. tecta*—5.88 %
D1—subresidents	27 species	F1—accidents	28 species
*Mammal nests*			
D5—eudominants	*Ph. borealis*—41.96 %	F5—euconstants	0
D4—dominants	*Ph. rackei*—18.58 %	F4—constants	*Ph. borealis*—42.92 %
D3—subdominants	*Ol. minima*—8.71 %		*Ph. rackei*—36.48 %
D2—residents	*Ph. advena*—6.76 %	F3—subconstants	*N. brevinguiculata*—25.75 %
	*Oo. karawaiewi*—5.48 %		*Oo. karawaiewi*—21.89 %
	*N. brevinguiculata*—5.26 %		*Ol. minima*—18.45 %
	*Oo. ovalis*—4.25 %	F2—accesory species	*Oo. ovalis*—14.16 %
D1—subresidents	24 species		*U. orbicularis*—14.16 %
			*Dis.* *modesta*—8.58 %
			*Din. perforatus*—8.15 %
		F1—accidents	22 species
*Bird nests*			
D5—eudominants	*O. orbicularis*—35.89 %	F5—euconstants	0
D4—dominants	*A. infirmus*—18.85 %	F4—constants	0
	*Uro. pyriformis*—17.94 %	F3—subconstants	0
D3—subdominants	*N. brevinguiculata*—14.40 %	F2—accesory species	*O. orbicularis*—9.69 %
D2—residents	*Al. flagelliger*—3.84 %		*A. infirmus*—7.30 %
	*U. orbicularis*—3.02 %		*N. brevinguiculata*—5.98 %
D1—subresidents	23 species	F1—accidents	26 species
*Ant hills*			
D5—eudominants	*Oo. spatulifera*—79.79 %	F5—euconstants	*Oo. spatulifera*—52.38 %
D4—dominants	0	F4—constants	0
D3—subdominants	*Tr. coccinea*—12.51 %	F3—subconstants	*Tr. coccinea*—16.67 %
D2—residents	*Din. woelkei*—3.67 %	F2—accesory species	*Oo. ovalis*—14.16 %
D1—subresidents	9 species		*Uro. pyriformis*—9.52 %
			*T. aegrota*—7.14 %
		F1—accidents	7 species

#### Tree holes

In tree holes from different tree species there were 34 species of Uropodina (Table 2). Similarly to dead wood, in the tree holes the most numerous and most frequent species was *Oo. ovalis*. *Uro. pyriformis* was slightly less numerous and frequent; three species (incl. *Dis. baloghi*) exceeded 55 % of the whole community (Table 6). *Oplitis alophora,* which is another very rare species in Poland, was found only in this microhabitat.

#### Mammal nests

Most of the analysed material comes from mole nests (*Talpa europea*). Thirty Uropodina species inhabited mammal nests (Table 2). The most frequent and numerous species were two species typical for this microhabitat, *Ph. borealis* and *Ph. rackei*; they constituted ca. 60 % of the entire community. *Phaulodiaspis rackei* could be also accidentally found in soil. Moreover, *N. breviunguiculata*, *Oo. karawaiewi* and *Ol. minima* were also frequent, but less numerous. *Uros. hunzikeri*, which is a very rare species, was found in the mole nests.

#### Bird nests

In the nests of almost 30 bird species (Błoszyk et al. 2006a), 28 species of Uropodina were found (Table 2). *L. orbicularis* was preponderant in the community, but also *A. infirmus*, *Uro. pyriformis,* and *N. breviunguiculata* were quite numerous (Table 6). These four species constituted >87 % of the community. However, the frequency of these species was low and did not exceed 10 %. The species found only in bird nests is *N. floralis*.

#### Ant hills

In the material from the ant hills (*Formica* s.l.), 12 uropodine species were found (Table 2). The most numerous (80 %) and most frequent (52 %) species was *Oo. spatulifera*. Also *Tr. coccinea* and *Oo. ovalis* occurred apparently frequently (Table 6).

### Role of merocenoses in ecosystem biodiversity

Table 7 shows the communities of Uropodina found in mole nests and in the soil samples, on the same meadow, near Jarocin (Wielkopolska). Out of the 11 species found in the mole nests, the two most dominant species (*Ph. rackei* and *Ph. borealis*) were not found in the soil. Morover, six species from the soil were not found in the nests. The average number of mites per sample volume was 30 times higher in the nests than in the soil. The frequency of all species occurring in both environments was always higher in the mole nests.

**Table 7:** Dominancy (D%) and frequency (F%) of Uropodina in mole nests and in soil of one meadow in Jarocin (Wielkopolska)

Species	Nests			Soil		
	Total	D%	F%	Total	D%	F%
*Ph. rackei*	360	39.52	32.35			
*Ph. borealis*	302	33.15	44.12			
*Ol. minima*	68	7.46	35.29	29	25.89	10.40
*N. breviunguiculata*	54	5.93	11.76	39	34.82	12.00
*Oo. ovalis*	48	5.27	20.59	1	0.89	0.80
*Oo. karawaiewi*	27	2.96	26.47	18	16.07	5.60
*Din. perforatus*	21	2.31	8.82	2	1.79	1.60
*Uro. orbicularis*	20	2.20	5.88	4	3.57	3.20
*Din. carinatus*	6	0.66	5.88	1	0.89	0.80
*Dis. modesta*	4	0.44	5.88	8	7.14	2.40
*Ur. tecta*	1	0.11	2.94	1	0.89	0.80
*Cilliba rafalskii*				2	1.79	0.80
*Din. inermis*				3	2.68	2.40
*Ne. splendida*				1	0.89	0.80
*Pr. punctatus*				1	0.89	0.80
*T. aegrota*				1	0.89	0.80
*Ur. pannonica*				1	0.89	0.80
Total	911			112		
Average no. of specimens per sample	26.79			0.90		
No. of samples	34			125		

In the 1,259 samples (407 from dead wood, 852 from soil and litter of horn-beam forests) collected in the three nature reserves in Wielkopolska, 33 species of Uropodina were found: 28 species in dead wood and 20 species in soil and litter (Table 8). Five species of Uropodina could be identified as typical soil species (*I. penicillata*, *Ol. misella*, *Ps. calcarata*, *Ur. pannonica*, *Uro. orbicularis*), whereas 13 species (*Uro. obovata*, *A. infirmus*, *Tre. elegans*, *Din. arcuatus*, *L. orbicularis*, *Tr. coccinea*, *Pseudouropoda* sp., *P. cylindricus*, *Ps. tuberosa*, *C. erlangensis*, *Dis. baloghi*, *N. breviunguiculata*, and *N. stylifera*) were found only in the material from the dead wood (Table 8). Only 15 species were present in both environments. The mite communities inhabiting dead wood or soil and litter had a different structure of dominancy. In the analysed soil and litter, the most numerous species were *T. aegrota* and *Ol. minima*, whereas in the dead wood *Oo. ovalis* and *Uro. pulchella* were more numerous and frequent. In both environments the specimens of these species constituted >50 % of the whole community.

**Table 8:** Dominancy (D%) and frequency (F%) of Uropodina in dead wood and soil and litter samples of horn-beam forests from natural reserves in Wielkopolska

Species	Dead wood			Soil and litter		
	Total	D%	F%	Total	D%	F%
*Oo. ovalis*	**2,806**	**71.29**	**56.51**	**915**	**22.25**	**27.11**
*Uro. pulchella*	**235**	**5.97**	**11.55**	13	0.32	0.94
*Ol. minima*	**203**	**5.16**	**14.00**	**917**	**22.30**	**32.04**
*Din. woelkiei*	**177**	**4.50**	**6.39**	19	0.46	0.35
*T. aegrota*	172	4.37	16.71	**1,184**	**28.79**	**38.97**
*Din. carinatus*	101	2.57	7.13	9	0.22	0.82
*Ur. tecta*	74	1.88	6.88	**874**	**21.25**	**34.39**
*T. pauperior*	26	0.66	3.69	61	1.48	2.46
*A. infirmus*	25	0.64	0.49			
*Po. sansonei*	18	0.46	1.23	1	0.02	0.12
*Uro. obovata*	16	0.41	0.98			
*Tre. elegans*	14	0.36	1.97			
*Din. arcuatus*	12	0.30	1.47			
*Din. perforatus*	11	0.28	1.47	3	0.07	0.35
*C. rafalskii*	9	0.23	0.98	44	1.07	2.00
*Din.* sp.	8	0.20	0.49	8	0.19	0.82
*L. orbicularis*	7	0.18	1.23			
*C. cassideasimilis*	7	0.18	0.74	17	0.41	0.82
*Tr. coccinea*	4	0.10	0.25			
*Pseudouropoda* sp.	3	0.08	0.74			
*P. cylindricus*	1	0.03	0.25			
*Ps. tuberosa*	1	0.03	0.25			
*C. erlangensis*	1	0.03	0.25			
*Uro. pyriformis*	1	0.03	0.25	1	0.02	0.12
*D. cordieri*	1	0.03	0.25	10	0.24	0.47
*Dis. baloghi*	1	0.03	0.25			
*N. breviunguiculata*	1	0.03	0.25			
*N. stylifera*	1	0.03	0.25			
*I. penicillata*				1	0.02	0.12
*Ol. misella*				2	0.05	0.12
*Ps. calcarata*				1	0.02	0.12
*Ur. pannonica*				31	0.75	2.35
*Uro. orbicularis*				2	0.05	0.12
Total	3,936			4,113		
Average no. of specimens per sample	9.67			4.83		
No. of samples	407			852		

Bold—dominat species

Five out of the seven most numerous species (*Oo. ovalis*, *T. aegrota*, *Ol. minima*, *Uro. pulchella*, and *Ur. tecta*) in both environments revealed a significant preference for each of the two types of environments (i.e., occurred more numerously; Table 9). The abundance of the uropodine mites in the samples from the dead wood is much (2 times) higher than in the soil samples.

**Table 9:** Mean (±SE) abundance of the seven most dominant Uropodina species in soil and dead wood in the three nature reserves of Wielkopolska

Species	Dead wood	Soil and litter	z^a^	*P*
*T. aegrota*	2.46 ± 2.86	3.57 ± 6.15	6.71	<0.001
*T. pauperior*	1.73 ± 0.70	2.91 ± 4.60	0.35	>0.05
*Oo. ovalis*	12.20 ± 20.44	3.96 ± 6.51	10.33	<0.001
*U. tecta*	2.64 ± 3.05	2.98 ± 5.39	7.91	<0.001
*Ol. minima*	3.56 ± 4.09	3.36 ± 3.71	5.17	<0.001
*Uro. pulchella*	5.00 ± 5.93	1.63 ± 1.06	3.06	<0.01
*D. woelkei*	6.81 ± 9.46	6.33 ± 7.51	1.73	>0.05

^a^Mann–Whitney U test

## Discussion

Błoszyk et al. have emphasized many times the specificity of Uropodina communities (Błoszyk and Olszanowski 1986; Błoszyk and Miko 1990; Błoszyk and Athias-Binche 1998; Błoszyk 1999; Błoszyk and Bajaczyk 1999; Skoracka et al. 2001; Bloszyk et al. 2003a, b, 2005a, 2006a; Bajerlein et al. 2006; Błoszyk and Gwiazdowicz 2006, and Gwiazdowicz et al. 2006). Also other researchers have provided cogent evidence for the specificity of zoocenoses of Uropodina in such microhabitats (e.g. Athias-Binche 1977a, b; Krištofík et al. 1993; Gwiazdowicz et al. 2000; Gwiazdowicz and Sznajdrowski 2000; Mašán 2001; Gwiazdowicz and Klemt 2004; Gwiazdowicz and Kmita 2004).

The uropodine species found in the soil and litter contain 92 % of all species found in Poland (Napierała 2008). The most characteristic feature of the uropodine mite communities inhabiting unstable microhabitats (such as dead wood, tree holes, mammal and bird nests, and ant hills) is not only their specific species composition but also their dominancy structure. The species composition differed among the merocenoses, more than in the communities occurring in soil and litter of different forest types. In each type of merocenose one or two of the dominant species constituted >50 % of the entire community, and some species were typical for a particular type (*Uro. pyriformis*, *Ph. borealis*, *Ph. rackei*, *Oo. spatulifera*, and *Tr. coccinea*). Instead of strong predomination of one species, soil communities often have a group of 4–5 species, which constitute their ‘core’. These are often the same species in each case, i.e., *T. aegrota*, *Ol. minima*, *Ur. tecta*, *Oo. ovalis,* and *Oo. karawaiewi*.

Uropodina species associated with soil and unstable microhabitats differ as to their reproductive strategies (Błoszyk and Olszanowski 1985a, b; Błoszyk 1999; Błoszyk et al. 2004). Communities of Uropodina inhabiting soil and litter are usually predominated by species which reproduce parthenogenetically (thelytoky) (e.g. *T. aegrota*, *Ur. tecta,* and *Ol. minima*), whereas in merocenoses bisexual species prevail (e.g. *Oo. ovalis*, and *Din. woelkei*). The only exception is *Uro. pulchella,* which is one of the most numerous species in dead wood, but it reproduces parthgenogenetically (male-to-female ratio 1:15) (Błoszyk et al. 2004). In this case, the number of the males rises proportionally to the increase of the population size (Błoszyk, unpublished data). For most of soil-inhabiting Uropodina (such species as *T. aegrota*, *T. pauperior*, *T. lamda*, *Ol. minima*, *U. orbicularis*), males are observed sporadically (Błoszyk and Olszanowski 1985b; Błoszyk 1999; Błoszyk et al. 2004, 2005b).

Uropodina species from soil and unstable microhabitats also have different modes of dispersion. The small size of merocenoses, their inconstancy, isolation and fragmentation compel such species to develop special ways of dispersion which will enable them to leave a disappearing habitat and find a new one. For most uropodine species passive dispersion between microhabitats is phoresy (Athias-Binche 1993, 1994; Błoszyk 1999; Bajerlein and Błoszyk 2004; Bajerlein et al. 2006; Błoszyk et al. 2006b). Soil, which is a more stable and homogenous environment, enables existence of a population consisting of clones of the paternal specimens, whereas unstable merocenose requires continual genetic recombination. The very low abundance of Uropodina in soil and problems in finding a sexual partner, force these mites to reproduce parthenogenetically (Błoszyk et al. 2004, 2005b).

The studies on the structure of Uropodina communities in merocenoses are important because they may shed new light on the issues concerning species composition of Uropodina in Europe after the regression of the last glaciation. Furthermore, merocenoses constitute ‘halts’ for the populations of many species, forming stepping stones in their dispersion. It is also possible that many Uropodina species have migrated from the South to the North of Europe because they were carried there by arthropods, birds, and mammals when the glacier regressed, and then they had to inhabit merocenoses. The colonization of soil and litter probably took place much later.

Unstable microhabitats enrich the overall biodiversity of forest and meadow ecosystems. Dead wood is one of the most important components in preserving biological diversity of forest ecosystems (Gutowski et al. 2002). The number of species that form Uropodina communities is proportional to the range and the number of microhabitats in a particular ecosystem. The presence of merocenoses increases the general biodiversity of an ecosystem—not only of Uropodina—therefore, it is important to protect them, e.g. by not removing dead wood from forests, not bricking up tree hollows, and leaving ant hills undisturbed.
